# Supplementary material for: Synthesis of Anion Exchange Membranes Containing PVDF/PES and Either PEI or Fumion®
Source: Membranes (Basel). 2022 Sep 30;12(10):959. doi: 10.3390/membranes12100959 (PMC9607123; doi:10.3390/membranes12100959)
Supplement: Supplementary file 1 [file membranes-12-00959-s001.zip › membranes-1802175-supplementary.pdf]

Synthesis of anion exchange membranes containing PVDF/PES and either PEI or Fumion®

Salazar-Gastélum, L.J.[1]; García-Limón, B.Y.[2]; Lin, S.W.[1]; Calva-Yañez, J.C.[1]; Zizumbo-López, A.[1]; Romero-Castañón, T.[3]; Salazar-Gastélum, M.I.[1,2] and Pérez-Sicairos, S.[1]*

[1]Tecnológico Nacional de México/Instituto Tecnológico de Tijuana/Centro de Graduados e Investigación en Química, Blvd. Alberto Limón Padilla, S/N Col. Otay Tecnológico, Tijuana, B. C. 22510, México.

[2]Tecnológico Nacional de México/Instituto Tecnológico de Tijuana/Departamento de Ingeniería Eléctrica y Electrónica, Blvd. Alberto Limón Padilla, S/N Col. Otay Tecnológico, Tijuana, B. C. 22510, México.

[3]Instituto Nacional de Electricidad y Energías Limpias, Ave. Reforma 113 Col. Palmira, Cuernavaca, Morelos, 62490, México.

*Correspondence: sperez@tectijuana.mx

Text S1. Cross-section of PEI membranes

The cross-section of membranes were observed using scanning electron microscopy (SEM, Tescan VEGA 3). Previous to analysis, all samples were sputter-coated with gold (99.999%) at 18 mA for 4 min using a SPI-MODULE sputter coater. The operating voltage was 15 KV and scales of 5 μm and 20 μm were used. Figures S1 and S2 show the micrographs of membranes based on PEI and Fumion® at 20 μm, where the complete cross-section of each membrane is observed. The upper section corresponds to the surface of the membranes exposed to the air during the solvent evaporation process and the lower section to the surface in contact with the glass plate. A general trend from higher to lower porosity is observed from the upper to the lower section, which is related to parameters of the preparation method such as thickness of the polymeric solution, chemical composition, solvent evaporation rate, among others.

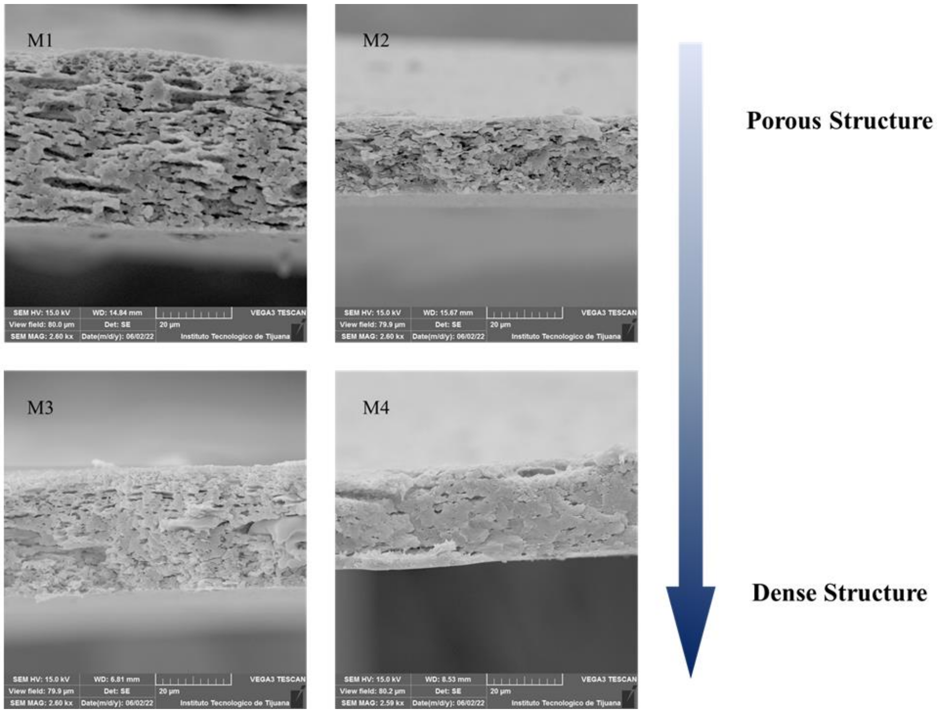

Figure S1. SEM images of cross section of PEI-based membranes (M1, M2, M3 and M4).

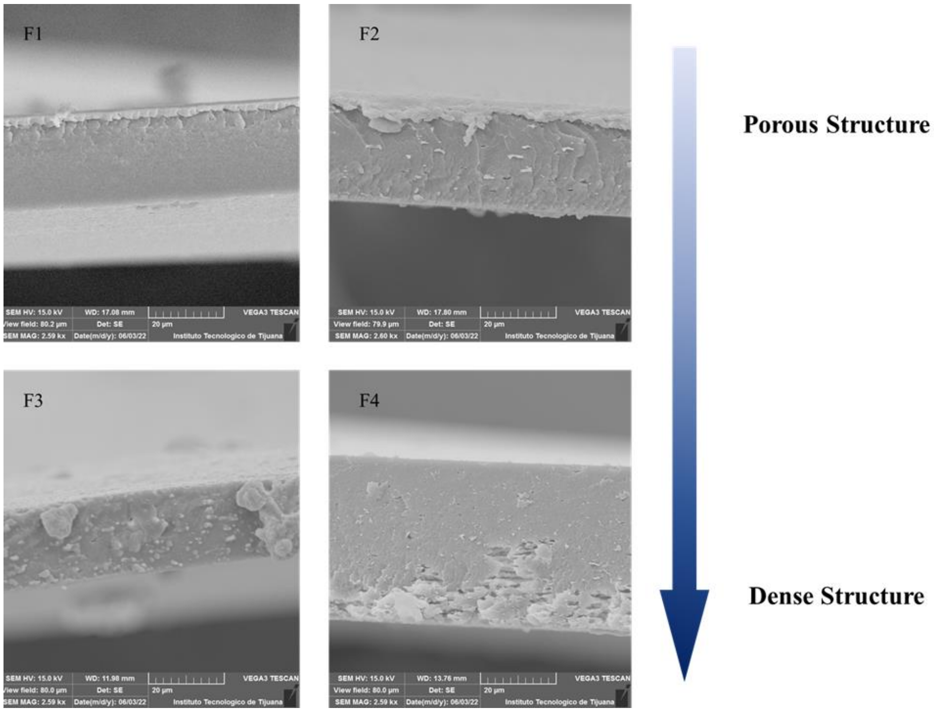

Figure S2. SEM images of cross section of Fumion®-based membranes (F1, F2, F3 and F4).
